# Genetic Variation in Seed Size in an Introgression Line Population of Upland Cotton

**DOI:** 10.3390/plants15111729

**Published:** 2026-06-03

**Authors:** Savyata Kandel, Linghe Zeng, Jane Dever, Carol Kelly, Derek Whitelock, Jinfa Zhang

**Affiliations:** 1 Department of Plant and Environmental Sciences, New Mexico State University, 945 College Drive, Las Cruces, NM 88003, USA; savuknde@nmsu.edu; 2 Crops Genetics Research Unit, Agricultural Research Service, United States Department of Agriculture, Stoneville, MS 38766, USA; 3 Pee Dee Research and Education Center, Clemson University, Florence, SC 29505, USA; jkdever@clemson.edu; 4 Texas A & M Agrilife Research and Extension Center, Lubbock, TX 79403, USA; 5 Southwestern Cotton Ginning Research Laboratory, Las Cruces, NM 88005, USA; derek.whitelock@usda.gov; 6 School of Agriculture, Tennessee Tech University, Cookeville, TN 38505, USA

**Keywords:** cottonseed index, fuzzy, acid-delinted seed, introgression, correlation

## Abstract

Upland cotton is an important fiber and oilseed crop. Cottonseed size, measured by seed index, is an important seed quality trait that affects seed germination, seedling vigor, fiber yield, and cottonseed nutrient content. However, genetic variation in cottonseed size is highly limited within upland cotton, limiting the genetic gain in cottonseed size. Introgression breeding can alleviate this bottleneck effect by introducing desirable genes from pima to upland cotton. This study was conducted to analyze the seed size from both fuzzy and acid-delinted seeds and to assess the appropriate cottonseed size. In 2022, a population of 1600 cotton introgression lines (ILs) was grown at Leyendecker Plant Science Center, NMSU, while three field tests were conducted in 2023, including NM with all the ILs and MS and TX each with 1000 ILs. The analysis of variance of seed size showed that genotypic and environmental variation were found in both types of seeds. The acid-delinted and fuzzy cottonseeds had a mean seed index of 9.58 g and 11.26 g, while the broad sense heritability was 0.56 and 0.32, respectively. Furthermore, the seed index was not significantly correlated with cottonseed oil and different fatty acids.

## 1. Introduction

Cotton (*Gossypium* spp.), one of the major cash crops, produces fiber and seeds. Cottonseed, a unit of reproduction, is a vital source of food, feed and biofuel. The seed development involves the coordinated growth of zygotic tissues, which includes the embryo, endosperm and maternal components (e.g., eggs) that determine the seed size and weight [1]. The seed development is completed into two phases: the morphogenesis phase includes cell division, endosperm and embryo development as well as cotyledon differentiation, whereas the maturing phase involves embryo development, seed dehydration, and source collection [2]. The seed coat epidermis is developed into cellulose-enriched mature fibers as single-celled hair, while the embryo of cottonseed synthesizes oils and proteins [3]. Therefore, both maternal and filial cottonseed tissues are economically significant.

Increasing yield is one of the major goals in plant breeding. The seed size and seed number are vital determinants of crop yield [4,5]. Therefore, the improvement in seed size has been a major concern of plant breeding since the domestication of crops. However, enhancing the seed size without affecting seed number through conventional plant breeding has allowed limited progress due to the trade-off between those yield components [6]. The seed size is an important factor during crop growth, harvesting, and processing [7]. As compared to small seeds, studies have shown that high-density cottonseeds have a higher chance of germination, seedling survival, growth, and oil content [8,9] and have stronger and longer fibers and higher fiber length uniformity but decreased micronaire [10,11]. Large-size seeds have increased seed fullness, dry matter weight per plant, and leaf area [12]. A positive correlation was reported between the seedling vigor and seed size in cotton [13]. The individual seed mass, along with total seed nutrient content, can predict early seedling vigor [13]. In general, large-seeded crops produce vigorous plants, which compete better for natural resources than small-seeded species [14,15]. This was also reported by Edmisten, where larger plant stands were obtained from larger seeds [16].

The cotton fiber is strongly controlled by seed development, as the fiber is a part of the seed. For example, larger seeds have a high sink strength to mobilize nutrient resources to the filial tissues, resulting in higher and longer cotton fibers [17,18]. In addition, larger seeds have more fibers per seed, as the surface area of the epidermis determines the number of fibers per seed [17]. It was found that only 25–30% of ovule epidermal cells develop into fiber cells at anthesis [17]; therefore, increasing the fiber proportion from the ovule epidermis may be an attractive technique to yield higher fibers [19]. However, an increase in seed numbers instead of individual seed size in a boll will most likely produce higher fiber cells per boll, because more seeds will have higher ovule surface areas for fiber initials. Indeed, both tetraploid and diploid cotton species show a strong correlation between the seed number and fiber production [17]. This may be achieved through either an increase in ovule number or a decrease in seed abortion. In fact, the number of seeds per boll and bolls per unit land area affects the cotton yield [20], because a higher number of small seeds per boll may have a larger surface area than a smaller number of large seeds per boll [21].

Over the last four decades, genetic gains in lint yield in the US were achieved through increased lint percentage, which was accompanied by a decreased seed size [11,22]. A negative association was observed between the cottonseed size and lint yield, because smaller seeds are associated with a higher lint percentage [13]. However, small cottonseed has two major problems in cotton production and processing. First, smaller cottonseed usually has a lower seed vigor [10]. The seed vigor issue can be addressed with the selection of denser or heavier seeds. Second, small cottonseed also causes trouble in post-harvest processing, as the small seeds can pass through the gin stand with lint making more seed coat fragments and causing the loss of seed and further problems in spinning and mechanical delinting [10]. However, growers prefer smaller seeds due to the increased number of seeds per pound reported by Edmisten, as they do not want to sacrifice yield for vigor, because cotton is primarily cultivated for fiber production [16]. Hence, a balanced cottonseed size is necessary to enhance the various sectors of the cotton industry.

Commercial upland cotton cultivars usually produce fuzzy seeds. However, the fuzz (also called linters) must be removed by acid-delinting, before the seeds can be used for planting. The acid-delinting process facilitates the flowing of seeds in modern planting equipment, which is restricted by linters and small amounts of long fibers that remain on the seed even after the ginning process. Those linters cause the cottonseed to clump together, making it difficult to achieve mechanical planting. There are two acid-delinting processes in use: wet acid and dry acid methods. The wet acid method uses either concentrated or dilute sulfuric acid. In this method, cottonseeds are allowed to pass through an acid bath, followed by subsequent neutralization and drying. While in the dry acid method, fuzzy seeds are placed in a sealed chamber where gaseous hydrochloric acid is pumped into the chamber and agitated. Then, the seeds are neutralized, as they do not require drying. The dry acid method is rarely used in areas having high relative humidity, as the air and seed moisture increase the heat generated by hydrochloric acid [10]. Each method requires specialized equipment, considerable time, and expense. Even though those delinting methods are very effective and inexpensive, there are some specific concerns with this process including worker safety, potential seed damage, deterioration of equipment, and waste disposal. As cottonseeds imbibe solvent, the physical properties of the seed coat may be altered, and some cottonseed may be lost by acid damage or during handling in the acid-delinting process. Thus, the integrity of a sample may be compromised. However, acid-delinted cottonseeds germinate faster and are less attacked by pathogens and accessible to mechanical planting.

Due to the limited genetic diversity among elite upland cotton (*G. hirsutum*), which results from extensive selection within the species to enhance the lint yield and fiber quality, opportunities for improving the seed size through intraspecific breeding are restricted [23]. To overcome this genetic bottleneck, *G. hirsutum* can be crossed with *G. barbadense*, followed by selection to improve the seed size. Interspecific introgression line populations derived from *G. hirsutum* and *G. barbadense* are frequently utilized to identify genomic regions associated with key quantitative traits in cotton [24]. *G. barbadense* serves as a valuable germplasm source for improving the yield [25], fiber quality [26], disease resistance, and oil content in *G. hirsutum* [27]. Consequently, the population of introgression breeding between these two species holds significant potential for enhancing the cottonseed size.

Most studies on cotton primarily focus on the fiber yield and quality, with comparatively less research on the seed quality [28,29,30]. However, the seed quality is an important factor in cotton stand establishment [31]; so, good quality seed is a crucial input to ensure high cotton yield with high economic benefits [7]. However, the lack of quality cottonseed may be seen as a relevant issue. Generally, the seed size is a broadly accepted measure of seed quality and a vital determinant of crop yield aside from seed numbers. As there is not much information available regarding the cottonseed size in previous works, this research provides the overall study of cottonseed size in a large introgression line population derived from a cross between upland and pima cotton. This population was previously used to analyze cottonseed oil and fatty acids [32]. So, this study is important to fulfill the gap in cottonseed size and provide awareness of its importance for higher fiber yield and seedling vigor. Therefore, this study was conducted to (1) analyze the seed size as measured by the seed index from both fuzzy and acid-delinted seeds in a large population of cotton introgression lines (ILs); (2) assess the appropriate seed size for both types of cottonseeds; (3) analyze the interrelationship among cottonseed oil, different fatty acids and seed index; and (4) group the cotton introgression lines into different clusters on the basis of seed index. The cluster analysis would be helpful to evaluate the diversity among cotton introgression lines for the seed index.

## 2. Results

The analysis of variance (Table 1) revealed the substantial variation among the studied cotton introgression lines for the acid-delinted seed index across environments. This variation in seed index among cotton genotypes and locations can be utilized for the improvement of seed size in cotton. In acid-delinted seeds, the average value for the 100-seed weight was 9.58 g. The values varied from 7.2 g to 12.2 g in Las Cruces, New Mexico, in 2022, and from 7.4 g to 13.8 g in Lubbock, Texas, in 2023. The overall seed index value ranges from 7.2 g to 13.8 g for acid-delinted cottonseeds (Table 2).

**Figure 1:**
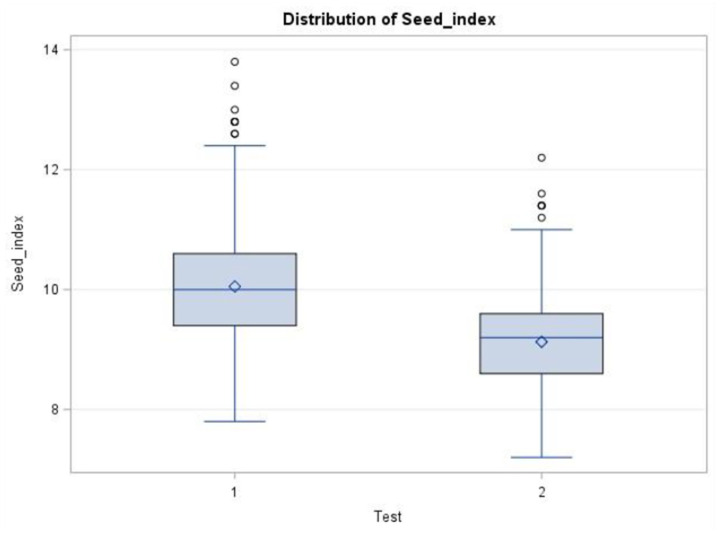
Box plot for seed index of cotton introgression lines of acid-delinted seeds in two locations (1: NM; 2: TX).

The average mean value for the acid-delinted seed index was 9.58 g with 7.16% of CV (Table 2). The intermediate seed size for acid-delinted cottonseed ranged from 9.16 to 10.14 g in our study. From the box plot (Figure 1), we found that the mean seed index was higher in cotton introgression lines from New Mexico as compared to Texas in acid-delinted seeds.

### 2.1. Variance Components and Broad-Sense Heritability of Seed Size for Acid-Delinted Cottonseeds

A larger environmental variance (0.4289) was found in comparison to the genotypic variance (0.2409) for the acid-delinted seed index. The residual variance was 0.3785 of the total phenotypic variances, indicating that unexplained variation, including experimental error and other uncontrolled factors, also contributed notably to the seed index variation. Furthermore, the broad-sense heritability was 0.56, suggesting that the seed index is under substantial genetic control, and the phenotypic selection for seed size in this population could be reasonably effective across environments.

ANOVA results revealed a substantial variation among the studied genotypes for the fuzzy seed index across environments. The mean value for the seed index was 11.26 g and ranged from 10 g to 14.4 g in Mississippi and 7.8 g to 12.8 g in New Mexico in 2023. High and significant location effects were also observed for the fuzzy seed index. The significant variation among cultivars and locations for the seed index indicated that the seed index (used as a measure of seed size) played an important role in the above variables (Table 3).

**Figure 2:**
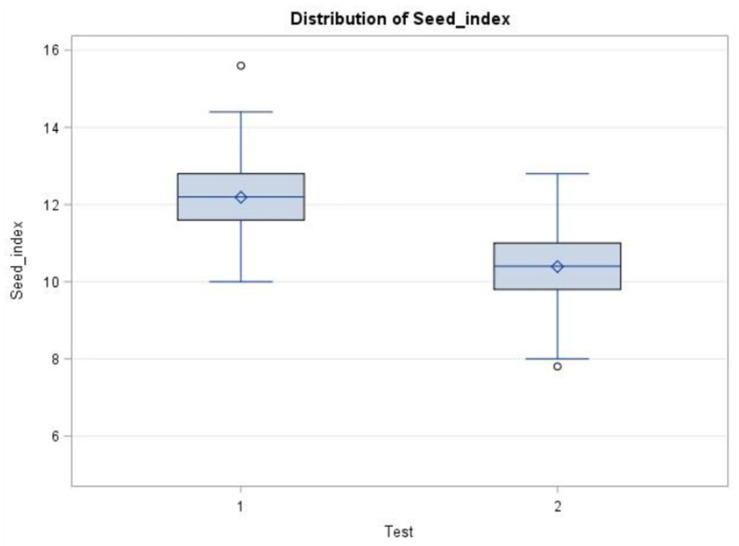
Box plot for seed index of 1000 cotton introgression lines of fuzzy seeds in two locations: 1 = Mississippi; 2 = New Mexico.

The average mean value of the fuzzy seed index was 11.26 g with an 8.66% coefficient of variance (Table 4). The intermediate seed index for fuzzy cottonseed ranged from 10.77 to 11.76 g in our study. From the box plot (Figure 2), we found that the mean fuzzy seed index was higher in cotton introgression lines from Mississippi, as compared to New Mexico, in fuzzy cottonseeds.

### 2.2. Variance Components and Broad Sense Heritability of Fuzzy Cottonseeds

The environmental variance (1.622) was higher than the genotypic variance (0.139), while the residual variance was 0.604. In addition, the broad-sense heritability was 0.32 for the fuzzy seed index. This relatively low heritability and large environmental variance suggest that the fuzzy seed index was highly influenced by environmental conditions, which may reduce the effectiveness of direct phenotypic selection across environments.

### 2.3. Hierarchical Cluster Analysis

**Figure 3:**
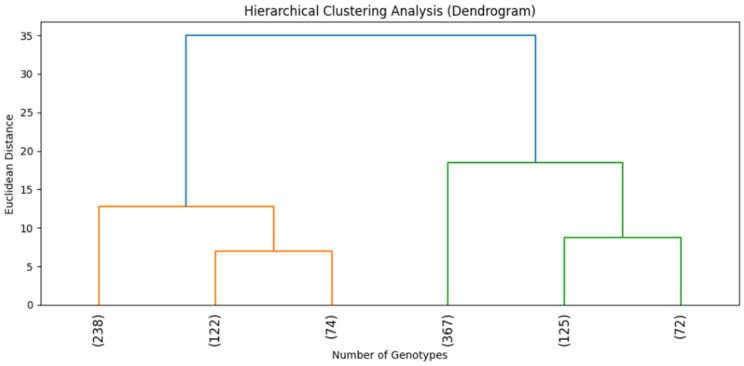
Dendrogram obtained from hierarchical cluster analysis on seed index of acid-delinted seeds.

Cluster analysis is one of the effective methods to establish a structured relationship among different genotypes. The ward method was used for the cluster analysis of seed index for acid-delinted cottonseeds from 1000 introgression lines in our study, as shown in Figure 3.

Based on the acid-delinted seed index, a population of cotton introgression lines was grouped into different clusters depending on the cut-off point. At five Euclidean distances, these introgression lines were grouped into six different clusters (Figure 3). Among six different clusters, clusters 4, 1, 5 and 2 are major clusters comprising 367, 238, 125 and 122 cotton introgression lines, respectively, while clusters 3 and 6 are smaller with 74 and 72 introgression lines, respectively (Figure 3). Genotypes included in one cluster were significantly different from genotypes grouped in another cluster regarding the mean seed index (Table 5).

Among six different clusters obtained from the hierarchical cluster analysis on acid-delinted seed index, cluster 6 has the highest average seed index, while cluster 3 has the lowest average seed index. The average seed index of all clusters is 9.59 g. This value is closer to the average seed index of cluster 4. So, selecting the cotton introgression lines from cluster 4 could be helpful to improve the lint yield, as well as the seed nutrient content of cottonseed. Aside from cluster 4, we can select the genotypes from cluster 1 as well, because it is comparatively closer to the intermediate value of acid-delinted seed index.

**Figure 4:**
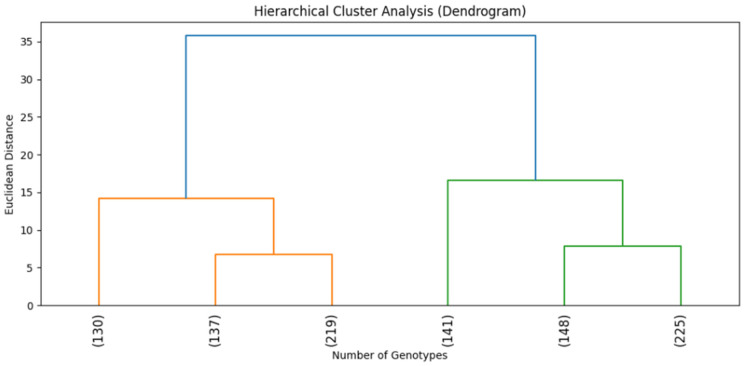
Dendrogram obtained from hierarchical cluster analysis on seed index of fuzzy seeds. The two line colors denote two different groups.

A population of 1000 cotton introgression lines was grouped into different clusters based on the fuzzy seed index at different cut-off points. At five Euclidean distances, those 1000 introgression lines were grouped into six different clusters (Figure 4). Among six different clusters, clusters 6 and 3 are major clusters comprising 225 and 219 introgression lines, respectively, while clusters 5, 4, 2, and 1 are comparatively smaller with 148, 141, 137, and 130 introgression lines, respectively (Figure 4).

Among the six clusters, cluster 4 has the highest average fuzzy seed index, while cluster 1 has the lowest average fuzzy seed index (Table 6). The mean value of the seed index of all clusters for fuzzy seeds was 11.28 g, which is closer to the average seed index of cluster 6. So, we can select the introgression lines from cluster 6 to consider the enhancement of both cotton fiber and seed nutrient traits. The average seed index value of cluster 3 is also closer to the intermediate value of the fuzzy seed index. Therefore, we can consider cluster 3, in addition to cluster 6, for the improvement of fiber and seed traits in cotton.

### 2.4. Correlation Among Oil, Fatty Acids and Seed Index

**Figure 5:**
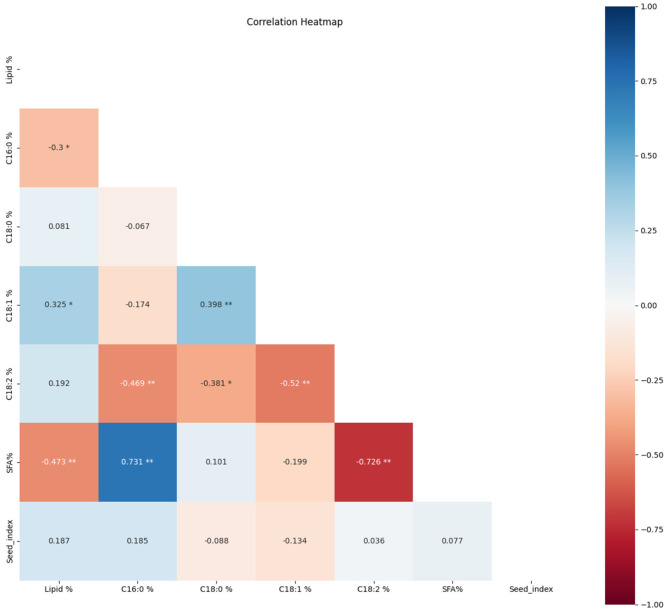
Correlation heatmap on lipid, fatty acids and acid-delinted seed index among cotton introgression lines. (SFA = saturated fatty acid; C18:2 = linoleic acid; C18:1 = oleic acid; C18:0 = stearic acid; C16:0 = palmitic acid). * and ** are significant at 0.05 and 0.01 significance levels, respectively. The gradient of blue explains the positive correlation, whereas red explains the negative correlation.

While analyzing the correlation between cottonseed oil, different fatty acids and acid-delinted seed index, no significant correlation was reported between the seed index, cottonseed oil, and fatty acids in this study (Figure 5). A highly significant and positive correlation (r = 0.73, *p* = 0.01) was found between saturated fatty acids (SFA) and palmitic acid (C16:0). In contrast, palmitic acid was negatively correlated with lipid (r = −0.30, *p* = 0.05). Likewise, SFA was significantly and negatively correlated with linoleic acid (r = −0.73, *p* = 0.01) and lipid (r = −0.47, *p* = 0.01) in cottonseed, whereas lipid was positively correlated with oleic acid (r = 0.32, *p* = 0.05).

## 3. Discussion

Genotypic differences were observed on the seed index in this study for both acid-delinted and fuzzy cottonseeds, which indicated that sufficient variability is present among the tested genotypes for this trait, and selection is possible for an improvement in seed size. This may be due to the large genetic variations among cotton introgression lines with respect to cottonseed size. In addition, there was a significant difference in the mean weight per 100 seeds between acid-delinted and fuzzy cottonseed. The acid-delinted and fuzzy cottonseeds had mean seed indexes of 9.58 g and 11.26 g, respectively. The fuzzy seed displayed a 17.54% increase in weight, which resulted from a combination of the residual linters on the seed. This is what would be expected from not removing the short fibers from the seed. Although counting fuzzy seeds requires more time than counting delinted seeds, the seed index of fuzzy seeds is the most time efficient, since it does not require delinting of the seeds. Furthermore, the acid-delinting process may compromise the integrity of seed samples due to the loss of seed fragments. However, the acid-delinted method removes all linters, reducing the possibility of seed clumping and providing a smooth seed coat, which improves the seed-to-soil contact and germination. Cleaner acid-delinted seeds have advantages of uniform planting, access to mechanical planting systems, and reduced disease attacks.

The seed index, a major yield factor, plays an inevitable role in increasing the cottonseed yield and seed nutrient content. Our research found a significant variation in seed size among cotton introgression lines developed from a cross between upland and pima cotton. This study confirmed the results observed in the previous literature. For instance, significant variations for the seed index were observed among the cultivars of *G. hirsutum,* while studying their yielding capacity [33,34,35,36]. In addition, a significant and positive correlation was found between the seed index and seed cotton yield while studying the performance of Egyptian cotton cultivars [37]. A significant variation in yield attributes among cultivars was observed while studying the seed index, yield and other yield-related traits in *G. hirsutum* [38,39]. The variation in seed size observed in the previous research might be due to genotypic and environmental differences, as well as the diverse genetic background of the breeding material used in various environmental conditions. In this study, the environmental variance was larger than the genotypic variance for acid-delinted and fuzzy cottonseed indexes. This indicates that environmental conditions substantially influenced the seed index expression. However, the moderate broad-sense heritability for acid-delinted seed index suggests that genetic factors still play an important role in determining the seed size, and effective genetic improvement through selection may be feasible. On the other hand, low broad-sense heritability for the fuzzy seed index implies that the environmental interactions associated with fuzzy seeds may have increased the environmental variability and reduced the precision of the genetic evaluation for the seed index.

In our study, the average seed index of acid-delinted cottonseeds was found to be higher in Las Cruces, New Mexico, in 2022, as compared to Lubbock, Texas, in 2023. These differences may reflect environmental variations between locations and years, including temperature conditions during boll and seed development, which can influence the accumulation and final seed size in cotton. Furthermore, the mean seed index of fuzzy cottonseeds was higher in Stoneville, Mississippi, in comparison with Las Cruces, New Mexico, in our research. These environmental differences may be associated with contrasting climatic conditions between locations. Stoneville has a humid subtropical climate with higher rainfall availability, which is suitable for cotton cultivation. On the other hand, Las Cruces is characterized by an arid climate with hot summers, where cotton is cultivated under irrigation. Environmental factors such as water availability and temperature during boll and seed development can influence the accumulation and final seed size in cotton. Therefore, the observed differences in the seed index among locations may partly reflect environmental effects on seed development.

In the hierarchical cluster analysis for both fuzzy and acid-delinted cottonseeds, around 1000 introgression lines were grouped into six different clusters at five Euclidean distances. The cotton introgression lines grouped into one cluster were significantly different from the introgression lines included in another cluster. The cluster analysis revealed substantial diversity among introgression lines for the seed size, suggesting potential opportunities for selecting complementary parental lines in breeding programs. Small-seeded genotypes had a higher number of seeds per pound, which gives a higher lint yield as compared to large-seeded cotton lines. This inverse relationship between seed size and seed number likely reflects a biological trade-off associated with assimilate partitioning during boll development. Genotypes with a higher number of seeds per boll may experience higher competition for available resources, resulting in smaller individual seed size. Large-seeded genotypes, on the other hand, had the highest fiber fineness, micronaire, and fuzz percentage. Understanding the balance between the seed size and seed number is important for breeding programs to optimize seed traits without negatively affecting yield components. Thus, genotypes with an intermediate seed size had several advantages including the highest ginning rate, highest fiber length, strength, and uniformity, as well as seed surface area and the highest number of seeds. Therefore, an intermediate seed size is needed to succeed in various sectors of the cotton industry, including higher fiber yields and increased cottonseed oil production. In our study, the intermediate seed size for acid-delinted ranged from 9.16 to 10.14 g, while fuzzy cottonseed ranged from 10.77 to 11.76 g.

The correlation analysis among the seed index, oil, and fatty acids in cotton showed a highly significant and positive correlation between saturated fatty acids (SFA) and palmitic acid. This refers to the fact that increasing the palmitic acid (C16:0) would significantly increase the SFA in cottonseed. In contrast, SFA was significantly and negatively correlated with oleic acid and lipid, suggesting that cottonseed having a higher SFA would decrease the oleic acid and lipid content. Recently, a similar correlation was found between SFA, lipid, oleic, and palmitic acid while analyzing the cottonseed oil and fatty acids through gas chromatography using a cotton introgression line population [32]. Otherwise, no significant correlation was reported between the seed index, cottonseed oil, and fatty acids in this study. This implies that there would be no effect on cottonseed oil and fatty acid content for the selection of the seed index or vice-versa. This finding was also in accordance with the recent results obtained from the analysis of cottonseed and lint traits in modern commercial transgenic cotton cultivars in regional high-quality tests [40]. Therefore, these relationships provide important insight into the feasibility of the simultaneous improvement in seed size and seed quality traits. Thus, understanding these associations is valuable for minimizing undesirable correlated responses during selection.

## 4. Materials and Methods

**Figure 6:**
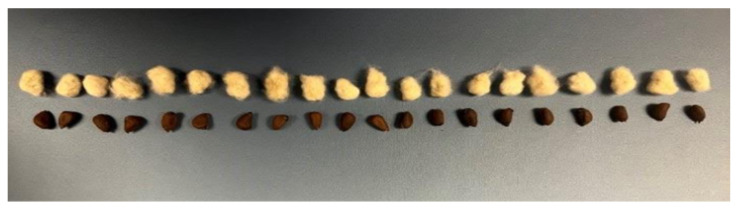
Fuzzy cottonseeds (top); acid-delinted cottonseeds (bottom).

The ILs used in this research were 10th generation progenies (F_10_) developed from a cross between Acala 1517-99 (*G. hirsutum*) and a pima cotton parent (*G. barbadense*) at New Mexico State University. A total of 1600 ILs were grown in Leyendecker Plant Science Center, NMSU in 2022. The experimental design was an augmented design with 27 blocks, and each block contained 4 checks (3 upland cotton and 1 pima including both parents) that were replicated in each block in a randomized complete block design. Twenty bolls per sample were harvested, and ginning was done to obtain fiber and fuzzy seeds. Because of the large size of the IL population, these 1600 ILs were randomly grouped into three different groups (Trial L, N, and T) each with 500–600 ILs. In 2023, field tests were conducted in three locations, including Las Cruces, NM, with all the ILs and Stoneville, MS, and Lubbock, TX, each with 1000 ILs including groups L and T. For this study of seed size, 1000 ILs from the L and T groups were used from NM, TX and MS in both 2022 and 2023. These ILs were arranged in an augmented design with one replication, and the four checks were included in each of 15–27 blocks depending on the testing locations. Seeds for each line were planted in a 1-row plot of 7.6 m (NM and TX) or 15.2 m (MS) long with a row-spacing of 1.02 m at a seeding rate of 10 seeds per meter using a plot planter in late April to early May in the three locations. Crop management practices, including weeding, irrigation, application of fertilizers, and pest controls, followed the local recommendations. At maturity, 20 open bolls were hand harvested from each plot for ginning and acid-delinting to obtain fuzzy and delinted seeds, respectively. Fuzzy seeds are cottonseeds that retain short fibers or fuzz (<0.5 cm) on their surface even after ginning. Acid-delinting helps to remove the short fibers or linters or fuzz from the surface of cottonseeds by treating those fuzzy seeds with sulfuric acid. The seed index (100-seed weight in grams) was measured by counting and weighing 50 seeds from both fuzzy and acid-delinted seeds (Figure 6). Although the direct measurement of 100 seeds is generally preferred, especially for fuzzy seeds, the same procedure was consistently applied across all samples and locations to maintain relative comparability among genotypes.

### Statistical Analysis

Data entry was carried out using Excel Microsoft 365. Variability in the seed size was measured by the analysis of variance (ANOVA). One thousand genotypes were used to estimate the effect of genotypes. A combination of locations and years was taken as an individual environment to determine the effect of the environment. As replicated check data were unavailable for the present analysis, the ANOVA was interpreted as an approximate assessment of variation among genotypes and environments rather than a fully replicated experimental analysis. This is the statistical limitation for this study. R-studio version 4.5.2 was used to perform ANOVA and estimate the variance components. In addition, a hierarchical cluster was analyzed for both acid-delinted and fuzzy seeds by using R-studio version 4.5.2. Pearson’s correlation analysis was analyzed to study the interrelationships among cottonseed index, oil, and different fatty acids. A subsample of these ILs was analyzed for the cottonseed oil and fatty acid contents [32].

## 5. Conclusions

Genetic variation existed among the studied cotton introgression lines for the seed index; so, selection is possible for an improvement in the cottonseed size. Environmental conditions such as temperature and water supply affect the size of cottonseed, as it also plays a major role in determining the seed size in addition to the genetics of cotton. The fuzzy cottonseed showed a 17.54% increase in weight as compared to acid-delinted seed, which resulted from a combination of the residual linters on the seed. The intermediate seed size in our research ranged from 9.16 to 10.14 g for acid-delinted seeds and 10.77 to 11.76 g for fuzzy cottonseeds. This range of seed size may be suitable to increase the cotton fiber yield and seed nutrient content, as well as to improve the seed quality traits. Otherwise, the seed index was not significantly correlated with cottonseed oil and fatty acids, suggesting that selection for the seed size does not affect cottonseed oil and fatty acids or vice versa. However, further research is needed for validation of the results obtained from this study.

## Figures and Tables

**Table 1:** Analysis of variance of seed index of acid-delinted seeds for 1000 cotton introgression lines at two locations (Las Cruces and Lubbock).

Source	df	Mean Square	F-Value	Pr > F
Genotype	977	1.0	2.05	<0.0001
Location	1	407.4	865.27	<0.0001
Residuals	977	0.5		

**Table 2:** Descriptive statistics for seed index of acid-delinted cottonseeds.

Grand Mean	Range	CV (%)
9.58	7.2–13.8	7.16

**Table 3:** Analysis of variance on seed index of fuzzy seeds for 1000 cotton introgression lines in two locations (Las Cruces and Mississippi).

Source of Variation	df	Mean Square	F-Value	Pr > F
Genotype	999	1.2	1.25	<0.0001
Location	1	1742.8	1830.70	<0.0001
Residuals	997	0.95		

**Table 4:** Descriptive statistics for seed index of fuzzy cottonseeds.

Grand Mean	Range	CV (%)
11.26	7.8–14.4	8.66

**Table 5:** Mean value of seed index for individual clusters obtained from hierarchical cluster analysis of acid-delinted cottonseeds.

Clusters	Number of Genotypes	Average Seed Index (g)
1	238	9.26
2	122	8.87
3	74	8.40
4	367	9.78
5	125	10.31
6	72	10.92

**Table 6:** Mean value of seed index for individual clusters obtained from hierarchical cluster analysis of fuzzy cottonseeds.

Clusters	Number of Genotypes	Average Seed Index (g)
1	130	10.24
2	137	10.71
3	219	11.06
4	141	12.38
5	148	11.84
6	225	11.45

## Data Availability

The original contributions presented in this study are included in the article.
